# Next-Generation and Single-Cell Sequencing Approaches to Study Atherosclerosis and Vascular Inflammation Pathophysiology: A Systematic Review

**DOI:** 10.3389/fcvm.2022.849675

**Published:** 2022-03-28

**Authors:** Liam W. McQueen, Shameem S. Ladak, Riccardo Abbasciano, Sarah J. George, M-Saadeh Suleiman, Gianni D. Angelini, Gavin J. Murphy, Mustafa Zakkar

**Affiliations:** ^1^Department of Cardiovascular Sciences, Clinical Science Wing, Glenfield Hospital, University of Leicester, Leicester, United Kingdom; ^2^Bristol Heart Institute and Translational Biomedical Research Centre, Bristol Medical School, Bristol Royal Infirmary, University of Bristol, Bristol, United Kingdom

**Keywords:** vascular inflammation, atherosclerosis, single-cell sequencing, next-generation sequencing, systematic review

## Abstract

**Background and Aims:**

Atherosclerosis is a chronic inflammatory disease that remains the leading cause of morbidity and mortality worldwide. Despite decades of research into the development and progression of this disease, current management and treatment approaches remain unsatisfactory and further studies are required to understand the exact pathophysiology. This review aims to provide a comprehensive assessment of currently published data utilizing single-cell and next-generation sequencing techniques to identify key cellular and molecular contributions to atherosclerosis and vascular inflammation.

**Methods:**

Electronic searches of Cochrane Central Register of Controlled Trials, MEDLINE, and EMBASE databases were undertaken from inception until February 2022. A narrative synthesis of all included studies was performed for all included studies. Quality assessment and risk of bias analysis was evaluated using the ARRIVE and SYRCLE checklist tools.

**Results:**

Thirty-four studies were eligible for narrative synthesis, with 16 articles utilizing single-cell exclusively, 10 utilizing next-generation sequencing and 8 using a combination of these approaches. Studies investigated numerous targets, ranging from exploratory tissue and plaque analysis, cell phenotype investigation and physiological/hemodynamic contributions to disease progression at both the single-cell and whole genome level. A significant area of focus was placed on smooth muscle cell, macrophage, and stem/progenitor contributions to disease, with little focus placed on contributions of other cell types including lymphocytes and endothelial cells. A significant level of heterogeneity exists in the outcomes from single-cell sequencing of similar samples, leading to inter-sample and inter-study variation.

**Conclusions:**

Single-cell and next-generation sequencing methodologies offer novel means of elucidating atherosclerosis with significantly higher resolution than previous methodologies. These approaches also show significant potential for translatability into other vascular disease states, by facilitating cell-specific gene expression profiles between disease states. Implementation of these technologies may offer novel approaches to understanding the disease pathophysiology and improving disease prevention, management, and treatment.

**Systematic Review Registration:** https://www.crd.york.ac.uk/prospero/display_record.php?ID=CRD42021229960, identifier: CRD42021229960.

## Introduction

Atherosclerosis of the arteries remains the leading cause of morbidity and mortality worldwide (1, 2). Several risk factors such as hyperlipidemia, smoking, hypertension and diabetes have been implicated in facilitating atherogenesis through activation of the inflammasome (3). LDL accumulated in sub-endothelial areas of arterial bends and branches can be oxidized (oxLDL), activating endothelial cells (ECs), and initiating the recruitment of monocytes. These monocytes subsequently differentiate into macrophages and endocytose oxLDL, which accumulates in these cells leading to foam cell formation. Foam cells cannot process oxLDL and can rupture if large amounts accumulate inside them. This can lead to the deposition of more oxLDL into the artery wall triggering more inflammatory reactions and thus completing a vicious cycle (4, 5). EC activation by modified lipids can induce the expression of adhesion molecules, chemokines, and cytokines leading to monocytes recruitment from the blood stream which is a key event in the development of atherosclerosis (6). Several genetic deletion studies have demonstrated that inflammation is required for lesion formation. Moreover, vascular inflammation can also contribute to the development of thrombotic complications of atheroma as activated macrophages can produce proteolytic enzymes that degrade collagen and thereby alter the structure of the fibrous cap (7–9).

Over the years, many methods have been established to study the development and progression of vascular inflammation and atherosclerosis. The recent development and utilization of next-generation and single-cell sequencing techniques are additional tools that will improve our understanding of complex diseases including atherosclerosis progression (10, 11).

This review aims to provide a narrative synthesis of published literature that utilize single-cell and next-generation sequencing methodologies to explore key molecular and cellular targets related to atherosclerosis and vascular inflammation onset and progression, as well as determining the broader utility and translatability of these methodologies.

## Methods

This systematic review was performed following guidance from the Preferred Reporting Items for Systematic Reviews and Meta-Analyses (PRISMA) statement standard (12). A study protocol was designed which conformed to the PRISMA protocol standard (13) and was registered at the International Prospective Register of Systematic Reviews (14).

### Study Eligibility

The inclusion criteria were: (1) Any studies utilizing single-cell and/or next-generation sequencing methodologies to study vascular inflammation, atherosclerosis, or both; (2) Human or animal subjects demonstrating atherosclerosis and/or vascular inflammation (all species, all sexes); and (3) All study models (*in vivo, in vitro*, and *ex vivo*).

Exclusion criteria included: (1) No implementation of single-cell and/or next-generation sequencing methodologies; (2) Aortic, transplant, or neurological inflammation and/or atherosclerosis; (3) Vasculitis or other vascular conditions; (4) Genome-wide association studies or meta-analysis studies; and (5) Subjects with co-morbidities or non-healthy control conditions (6); Conference and meeting abstracts, case reports and literature reviews; (7) Studies not published in English.

### Data Sources and Search Strategy

Electronic searches were conducted using the Cochrane Central Register of Controlled Trials, MEDLINE, and EMBASE without date or language restriction from inception until February 2022. The search strategy employed to determine studies of relevance utilized combinations of keywords such as “scRNA-seq,” “single-cell sequencing,” “next-generation sequencing,” “vascular inflammation,” “vasculitis,” “atherosclerosis,” and “arteriosclerosis.” A full description of the search terms is listed in the Supplementary Materials. In addition, the reference lists of all retrieved articles were hand searched for further relevant studies not previously identified. Only papers that were published in English were considered for subsequent analysis. References from selected papers were additionally scanned for relevant articles to ensure the literature search was thorough. Reviewers L.W.M and R.A. performed the database searches.

Search results were imported into the Rayyan QCRI web app (15), and duplicates were identified and removed. To select relevant papers identified by the electronic search, papers were assessed initially by their title, then by analysis of their abstracts. Reviewers L.W.M., S.L. and M.Z performed this stage independently. Studies not excluded after this stage were then examined in full to assess their relevance. All authors then validated the final selected papers, and any discrepancies will be resolved by discussion.

### Data Extraction

A standardized form was developed to extract data from the included studies for assessment of study quality and evidence synthesis. This form was tabulated using Microsoft Excel 2016 (Microsoft, Redmond, Washington). Data extraction first considered data from figures, tables and graphs (using digital ruler software where appropriate), followed by data extraction from the main text. Data extracted to the standardized form were categorized under the following headings: author, title, year, journal, research aims and objectives, subjects examined, cell type examined, sequencing method, research methodology, and key findings. Author L.W.M. performed data extraction, all authors validated the findings, and any discrepancies were resolved by discussion.

### Study Outcomes

The primary outcome measure was to determine the contributions of single-cell and/or next-generation sequencing techniques in the field of atherosclerosis and/or vascular inflammation development and progression. The secondary outcome measure was to determine the utility and translatability of these findings and sequencing methodologies to other related vascular disease research.

### Bias and Quality Assessment

Quality of included studies was assessed using the ARRIVE guidelines checklist (16). For assessment of the risk of bias, SYRCLE, a modified version of the Cochrane Risk of Bias tool specifically for animal intervention studies, was utilized (17). Following data extraction, author L.W.M. performed quality and risk of bias assessment. Any discrepancies were resolved by discussion between all authors.

### Data Synthesis

A narrative synthesis of all included studies was performed, with all relevant data tabulated where appropriate. For all outcomes, data was extracted in text format, or as mean ± standard deviation for numerical values. Where applicable, continuous variables were summarized with standardized mean difference, and dichotomous data was summarized with risk and odd ratios. Where relevant data was missing, authors were contacted (where possible); otherwise, data was presented as described in the article or Supplementary Material. Given the anticipated diversity of outcome measures, limited scope for statistical analysis was expected and as such, meta-analysis was not undertaken.

## Results

**Figure 1:**
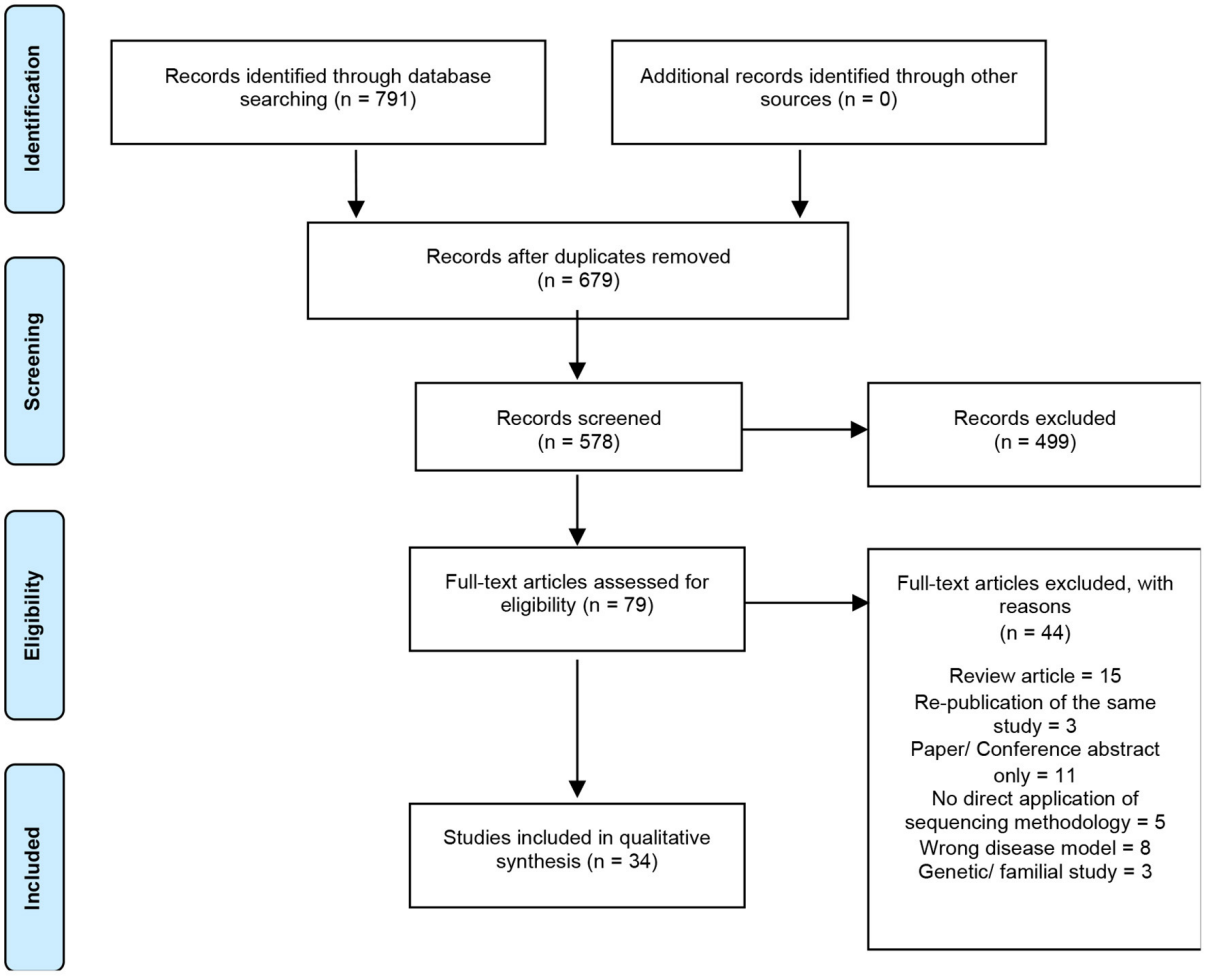
Summary of electronic literature search protocol. PRISMA flowchart detailing process of systematic literature searching, screening and selection (*n* = number of studies).

A total of 791 articles were identified by the search strategy, 679 following the removal of duplicates, which were then screened against the inclusion/exclusion criteria. Of these papers, only 34 were eligible for final synthesis (Figure 1). A summary of the characteristics of all included studies are reported in Table 1.

**Table 1:** Data characterization for included studies.

**References**	**Aims and objectives**	**Target**	**Subject examined**	**Sequencing methodology**	**Cell type examined**
Tang et al. (18)	Aim to determine the existence of Sca1+ vascular stem cells *in vivo*, and their role in vascular repair	Vessel repair	Mouse	Single-cell RNA-sequencing	Stem cells
Sharma et al. (19)	Understand whether Tregs are essential for the regression of atherosclerotic plaques, and if so, to identify key mechanisms by which Tregs contribute to plaque repair and contraction	Atherosclerotic regression	Mouse and cell culture	Single-cell RNA-sequencing	Lymphocytes
Pan et al. (20)	Understand SMC transdifferentiation during atherosclerosis and to identify molecular targets for disease therapy	SMC phenotypic switching	Mouse and human	Single-cell RNA-sequencing	Smooth muscle cells
Gu et al. (21)	Aim to perform scRNA-seq of aortic adventitial cells from WT and ApoE-deficient mice to explore their heterogeneity, diverse functional states, dynamic cellular communications, and altered transcriptomic profiles in disease	Adventitial transcriptome	Mouse	Single-cell RNA-sequencing	Adventitial cells
Cochain et al. (22)	Aim to determine and classify macrophage heterogeneity in both healthy and atherosclerotic aortas of mice using single-cell RNA-sequencing technology	Macrophage heterogeneity in atherosclerosis	Mouse and human	Single-cell RNA-sequencing	Macrophages
Kokkinopoulos et al. (23)	Aim to clarify the role of AdvSCA-1+ progenitor cells in native atherosclerosis, *via* elucidation of their differential gene expression profile between atherosclerosis-resistant and atherosclerosis-susceptible mice	Adventitial progenitor cells	Mouse	Single-cell RNA-sequencing	Stem cells
Rahman et al. (24)	Aim to investigate the source of, and functional requirement for, M2 macrophages in atherosclerosis regression, using a mouse aortic transplantation model	M2 macrophages in atherosclerosis regression	Mouse	Single-cell RNA-sequencing	Macrophages
Gu et al. (25)	Aim to further elucidate the role of perivascular adipose tissue (PVAT), with specific interest in characterizing the transcriptomic profile of PVAT-derived mesenchymal stem cells (PV-ADSCs) and their role in vascular remodeling	Adventitial cells in vascular remodeling	Mouse and cell culture	Single-cell RNA-sequencing	Adventitial cells and stem cells
Winkels et al. (26)	Aim to define an atlas of the immune cell landscape in atherosclerotic lesions, using single-cell RNA-sequencing and mass cytometry (cytometry by time of flight), *via* comparison of healthy and diseased arteries in mouse and human	Immune cells in atherosclerotic lesions	Mouse and Human	Single-cell RNA-sequencing	Lymphocytes
Wirka et al. (27)	Aim to determine: (1) which cell type(s) express Tcf21 during lesion development, (2) how does Tcf21 affect the phenotype of these cells, and (3) how does Tcf21 affect disease risk	SMC phenotypic switching	Mouse, cell culture, and human	Single-cell RNA-sequencing (CITE-Seq and ChIP-Seq) AND next-generation RNA-sequencing	Smooth muscle cells
Kim et al. (28)	Further investigate the specific effects of environment-sensing aryl hydrocarbon receptors (AHR) on the vascular SMC phenotype in atherosclerotic disease	SMC phenotypic switching	Mouse and cell culture	Single-cell RNA-sequencing AND next-generation RNA-sequencing (ChIP-Seq AND ATAC-Seq)	Smooth muscle cells
Kim et al. (29)	Aim to examine the transcriptomic profiles of foamy and non-foamy macrophages isolated from atherosclerotic intima, to determine their functional role and contribution to the disease	Transcriptome difference of foamy and non-foamy macrophages	Mouse and human	Single-cell RNA-sequencing AND next-generation RNA-sequencing	Macrophages
Steffen et al. (30)	Scrutinize the identity of sca1+/flk1+ cells, establish a phenotype for these cells, to amend the current hypothesis of vascular regeneration by circulating cells and gain understanding of their role in atherosclerotic disease	Vascular (endothelial) regeneration	Mouse	Next-generation RNA-sequencing	Stem cells
Mendez-Barbero et al. (31)	Aim to further elucidate the role of TWEAK/Fn14 in vascular remodeling, by identifying the downstream molecular mediators of this relationship, and how this has a functional effect on vascular smooth muscle cells (VSMCs)	SMC proliferation and migration	Mouse, cell culture, and human	Next-generation RNA-sequencing	Smooth muscle cells
Lai et al. (32)	Aim to explore the dynamic expression of EndMT genes in vascular endothelial cells under atheroprotective pulsatile shear stress and atheroprone oscillatory shear stress using RNAseq	Endothelial-to-mesenchymal transition	Mouse and cell culture	Next-generation RNA-sequencing	Endothelial cells
Karere et al. (33)	Aim to determine miRNA expression profile differences in baboons with low and high serum low-density lipoprotein cholesterol in response to diet. Aim to establish if any of these miRNAs are relevant to dyslipidemia and risk of atherosclerosis	MicroRNA relevance in dyslipidemia	Baboons	Next-generation microRNA-sequencing	Blood (micro RNAs in low/high LDL-C baboons with HCHF diet)
Depuydt et al. (34)	Aim to utilize single-cell transcriptomics and chromatin accessibility to gain a better understanding of the cellular heterogeneity and pathophysiology underlying human atherosclerosis	Atherosclerotic plaque composition	Human	Single-cell RNA-sequencing and single-cell ATAC-sequencing	Atherosclerotic plaques from carotid artery
Li et al. (35)	To study the role of macrophages and monocytes. In the CV system using a cell line model; to study the effect of matrix stiffness on macrophages behavior in atherosclerosis; to determine the synergistic role of ox-LDL and matrix stiffness on macrophage behavior, such as migration, inflammation, and apoptosis	Matrix stiffness on macrophage behavior (inflammation)	Cell culture	Next-generation microRNA-sequencing	Macrophages
Lin et al. (36)	Aim to improve the understanding of the origins and fates of macrophages in progressing and regressing atherosclerotic plaques using a combination of single-cell RNA sequencing and mouse genetic fate mapping	Macrophage heterogeneity in atherosclerosis	Mouse	Single-cell RNA-sequencing	Macrophages
Alencar et al. (37)	Aim to further define SMC subsets within atherosclerotic lesions, with the goal of identifying factors and mechanisms that promote beneficial SMC phenotypic transitions as novel therapeutic targets	SMC phenotypic switching	Mouse and human	Single-cell RNA-sequencing, Next-generation RNA-sequencing and ChiP-Seq	Smooth muscle cells
Li et al. (35)	Aim to clarify the specific functions and regulatory mechanisms of macrophage subsets present in vascular inflammation and atherosclerosis	Macrophage heterogeneity in atherosclerosis	Human and cell culture	Next-generation microRNA-sequencing	Blood (exosome microRNAs effect on macrophages)
Wolf et al. (38)	Aim to interrogate the function of autoreactive CD4+ T cells in atherosclerosis, through the use of a novel tetramer of major histocompatibility complex II to track T cells reactive to the mouse self-peptide apo B978-993 (apoB+) at the single-cell level	Immune cells in atherosclerotic lesions (T-cells)	Mouse	Single-cell RNA-sequencing AND next-generation RNA-sequencing	Lymphocytes
Zhou et al. (39)	Aim to investigate how the endothelial glucocorticoid receptor regulates vascular inflammation	Regulation of vascular inflammation *via* endothelial glucocorticoid receptors	Mouse and cell culture	Next-generation RNA-sequencing and ChiP-Seq	Endothelial cells
Bao et al. (40)	Aim to identify the transcriptome and proteome of stable and unstable atherosclerotic plaques	Atherosclerotic plaque transcriptome and proteome (stable vs. unstable)	Human	Next-generation RNA-sequencing	Atherosclerotic plaques (stable vs. unstable)
Gallina et al. (41)	Aim to identify the mechanisms underlying vascular smooth muscle cell phenotypic transitions associated with atherosclerosis and vascular injury, with specific focus on the glutamate receptor signaling	SMC phenotypic switching	Mouse, rat, and human	Single-cell RNA-sequencing and next-generation RNA-sequencing	Smooth muscle cells
Jiang et al. (42)	Aim to investigate the identity and role of CD34+ cells in vascular regeneration	Vascular (endothelial) regeneration	Mouse	Single-Cell RNA-Sequencing	CD34+ progenitor cells
Kan et al. (43)	Aim to characterize the cellular heterogeneity and diverse functional states within the wall of the ascending aorta in healthy and diseased mice using scRNA-seq to better understand the etiology and progression of aortic disease in HFD-induced obesity	Cell composition of healthy and diseased arteries	Mouse	Single-cell RNA-sequencing	Healthy and diseased aortas
Li et al. (44)	Aim to determine the specific contributions of disturbed flow on the heterogeneity of cells within the affected arterial vasculature	Effect of disturbed flow (shear stress) in the cellular and molecular composition of carotid arteries	Mouse	Single-cell RNA-sequencing	Carotid arteries (under disturbed flow)
Liang et al. (45)	Aim to utilize scSeq to examine VSMC phenotype in carotid artery calcified plaque cores and surrounding tissue to determine phenotype switching markers and mechanisms	SMC phenotypic switching	Human	Single-cell RNA-sequencing	Smooth muscle cells
Lin et al. (46)	Aim to understand the origin and phenotypic heterogeneity of smooth muscle cells (SMCs) contributing to intimal hyperplasia, with specific focus on how vascular cells adapt to the absence of elastin (Eln)	SMC phenotypic switching	Mouse	Single-cell RNA-sequencing	Smooth muscle cells
Brandt et al. (47)	Aim to comprehensively characterize the transcriptomic profile of phenotypically modulated VSMCs and identified mediators of VSMC transdifferentiation and their link to plaque rupture in human atherosclerosis	SMC phenotypic switching	Mouse	Single-cell RNA-sequencing	Smooth muscle cells
Newman et al. (48)	Aim to identify which cells, factors and mechanisms contribute to the fibrotic cap formation in atherosclerotic lesions	Fibrous cap composition	Mouse	Single-cell RNA-sequencing and next-generation RNA-sequencing	Atherosclerotic plaques (fibrotic cap)
Quiles-Jimenez et al. (47)	Aim to clarify the specific function of DNA glycolase Neil3 in the development of atherosclerosis, specifically in regard to vascular smooth muscle cell phenotypic modulation.	SMC phenotypic switching	Mouse and cell culture	Next-generation microRNA-sequencing	Smooth muscle cells
Burger et al. (49)	Aim to identify heterogeneous leukocyte clusters with distinct atherosclerosis disease-relevant gene expression signatures and to unveil their role in atherosclerosis pathology	Resident macrophage function in atherosclerosis	Mouse	Single-cell RNA-sequencing	Macrophages + smooth muscle cells

**Figure 2:**
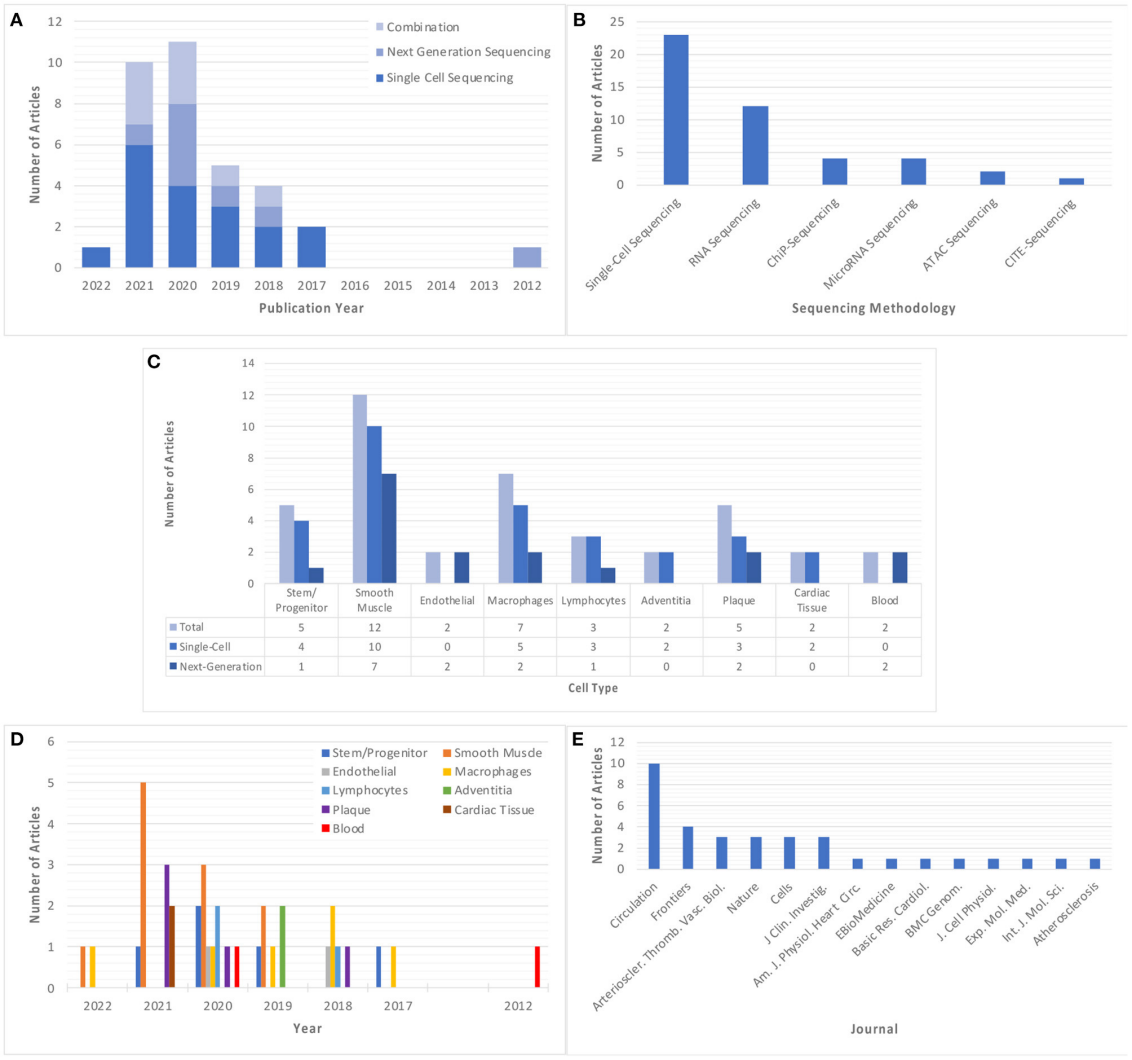
Summary of the main descriptors of included studies. **(A)** Year of publication of each included study, classified based on sequencing method. **(B)** Sequencing method of each included study. Where a study utilizes more than one sequencing method, this study is classified in each relevant group. **(C,D)** Cell type explored in each included study, classified based on cell/tissue type and sequencing methodology used, respectively. Note some studies explored multiple cell types and have been classified in all relevant groups. **(E)** Published journal for each included study. “Circulation” and “Nature” refer to all Circulation- or Nature-related journals which are encompassed by this publisher.

Studies using single-cell and/or next-generation sequencing to explore atherosclerosis and/or vascular inflammation appeared as early as 2012, however, there was an upsurge in the prevalence of these studies starting in 2017 (Figure 2A). The most employed sequencing methodology in these studies is single-cell sequencing (*n* = 23), which was first utilized in 2017 and features more frequently in subsequent years. Comparatively, next-generation sequencing was utilized less frequently (*n* = 12), with no significant trend in its use over time. A total of 10 articles utilized a combination of sequencing methods, with some articles using chromatin immunoprecipitation sequencing (ChIP-Seq) (*n* = 4), microRNA sequencing (*n* = 4), assay for transposase-accessible chromatin sequencing (ATAC-Seq) (*n* = 2) and cellular indexing of transcriptomes and epitopes sequencing (CITE-Seq) (*n* = 1) (Figure 2B). The most commonly studies cell types were smooth muscle cells (SMCs) (*n* = 10), followed by macrophages (*n* = 6) and stem/progenitor cells (*n* = 5), although these techniques are also applied more broadly to investigate the cellular and molecular characteristics of tissue, atherosclerotic plaque, and blood samples (Figure 2C). Interestingly, there appears to be a trend in the types of cells studied over time, with focus on smooth muscle cells almost tripling in prevalence from 2019 (*n* = 2) to 2021 (*n* = 5), whilst focus on macrophages has been consistent for a longer period (2017–2022), but at a lower prevalence (Figure 2D). All included studies were published in journals with impact factor ≥4, with the vast majority appearing in the publications Circulation and Circulation Research (*n* = 10) (Figure 2E).

### Cell-Specific Sequencing

#### Smooth Muscle Cells

Ten articles (20, 27, 28, 31, 37, 41, 45–47, 50) addressed the role of SMCs in atherosclerosis and vascular inflammation. Four of these articles (20, 45, 46, 50) utilized single-cell sequencing exclusively, one (31) utilized next-generation RNA sequencing exclusively, one (47) utilized next-generation micro-RNA sequencing, and the remaining four (27, 28, 37, 41) utilized a combination of these methodologies, including the use of ChIP-Seq (27, 28, 37), ATAC-Seq (28) and CITE-Seq (27).

Pan et al. (20) identified a novel transient cell state in mouse models related to SMC phenotypic switching using single-cell sequencing, which exhibited upregulation of markers *Ly6A, VCAM1*, and *Ly6C1*, attributed to stem, endothelial and mesenchymal cells respectively, accompanied by loss of SMC-specific markers. These cells, termed SEM cells, were the result of transdifferentiation of SMCs. lineage analysis revealed potential for SEMs to differentiate back to an SMC phenotype, or into a “fibrochondrocyte” or macrophage-like cell. Additionally, these cells did not exhibit traditional mesenchymal markers, suggesting a unique SMC-derived cell state, and this unique state was shown to exist in human atherosclerotic lesions.

Wirka et al. (27) undertook single-cell sequencing of SMC-specific lineage-traced mice to determine the fate of SMCs at baseline and after high-fat diet. Sequencing of aortic root atherosclerotic plaques revealed two SMC clusters with distinct gene expression profiles which become less defined as the disease progressed. This second, modulated cluster expressed markers of SMC differentiation such as *CNN1, FN1*, and *COL1A1*, and appeared to be more closely related to a fibroblast-like phenotype with expression of markers decorin and biglycan. These cells, termed fibromyocytes (FMCs), were localized to vessel medias, suggesting phenotypic switching occurs prior to migration into the plaque, and FMCs appeared to be absent of any markers associated with macrophages suggesting FMCs exist as a discrete species. The *TCF21* gene was shown to exert control over the fate of SMC phenotypes, with upregulation in progressive atherosclerotic burden, and association with GWAS data suggesting *TCF21* action on SMC phenotype modulation toward an FMC phenotype has an atheroprotective role.

Kim et al. (28), utilizing an established SMC-specific lineage-traced mice models (27), performed single-cell sequencing of atherosclerotic aortic root tissue to identify the role of aryl hydrocarbon receptors (AHRs) on affecting SMC phenotype. AHR expression was found to colocalise with the previously identified FMC cell cluster, as well as with *TCF21*, to the lesion intima and fibrotic cap, further validated using a combination of RNAScope and immunofluorescence. Sequencing analysis of AHR-knockout models revealed an increased population of FMCs in the lesion intima vs. wild-type (WT), as well as a distinct cell cluster with expression profiles related to ossification, collagen organization and *TGF*β signaling. Additionally, upregulation of factors specifically strongly related to chondrocytes were identified (e.g., *Sox9* and *Runx2*) in this cluster which the researchers termed “chondromyocytes” (CMC).

Mendez-Barbero et al. (31) undertook RNA sequencing of cultured murine vascular SMCs to identify the *TWEAK/Fn14* axis as a central regulator of SMC proliferation and migration in response to vascular injury. Comparison of gene expression between cells in the presence and absence of recombinant *TWEAK* (*rTWEAK*) treatment revealed upregulated expression of markers related to cell proliferation, migration, and motility in response to injury. The *TWEAK/Fn14* interaction was shown to downregulate cell cycle regulator *p15*^*INK*4*B*^ and upregulated cyclin-dependent kinase 4/6 and cyclin D1 *via* induced phosphorylation of *MAPK* extracellular signal-regulated kinases 1 and 2 (*ERK1/2*) and *AKT*, as well as *NF*κβ subunit *p65*. Conversely, the absence of *TWEAK* using mouse knockout models was shown to inhibit SMC proliferation and markedly reduce the neointimal area of mice with wire injury-induced vessel damage, suggesting a central role for *TWEAK/Fn14* in facilitating vascular remodeling.

Alencar et al. (37) utilized a combination of single-cell and next-generation RNA sequencing, combined with ChIP-Seq, to further define SMC phenotypes which affect the pathogenesis of atherosclerotic lesions. SMC^KLF4^ and SMC^OCT4^ KO and WT mice models were developed to validate the gene expression modulations of *Klf4* and *Oct4* in lesion development and stability. Next-generation sequencing and ChIP-Seq analysis identified contrasting lesion morphology phenotypes in each KO model, with *Oct4* targets enriched for genes involved in cell pluripotency and migration, whilst *Klf4* targets were enriched in genes involved in leukocyte recruitment and extracellular matrix (ECM) organization, suggesting an opposing role for these transcription factors in lesion phenotype. Single-cell sequencing of brachiocephalic artery lesions identified 14 distinct clusters of which seven were shown to derive from an SMC origin *via* lineage-tracing modeling, with these clusters lacking traditional SMC markers (e.g., *Myh11*) but expressing markers such as *Vcam1, Lgals3, Spp1*, and *Sox9* among others, suggesting significant cellular plasticity of cells during lesion development. The *Lgals*3^+^ cluster was identified as a chondrogenic-like state, which, in *Klf4* KO mice, was markedly reduced in line with a reduction in lesion size and fibrous cap thickness. *Lgals*3^+^ SMCs were shown to exist as a unique intermediate stem cell-like, ECM remodeling phenotype, representing up to two thirds of all lesion SMCs, which are further differentiated into several other pro-inflammatory and *Klf4* dependent osteogenic phenotypes which contribute to plaque calcification and destabilization.

Gallina et al. (41) investigated the mechanisms underpinning vascular SMC phenotypic switching specific to AMPA-type glutamate receptors shown to exert an effect on pulmonary vascular remodeling. Microarray transcriptome and single-cell sequencing revealed human atherosclerotic plaque samples contain relevant signaling and receptor components for glutamate turnover and signaling, specifically GRIA1 and GRIA2 which were exclusively detected in cells of mesenchymal (primarily SMC) origin. Expression of these transcripts were associated with phenotypic transition of SMCs, as determined by next-generation sequencing from a rat carotid artery injury and repair model at different timepoints. *Gria1* were significantly inversely correlated with traditional SMC marker expression (e.g., *Myh11*) in injured arteries, whilst *Gria2* was expressed at lower levels, more prevalent in non-injured vessels and had a positive correlation with SMC marker expression.

Liang et al. (45) undertook single-cell sequencing of carotid artery calcified cores and paracellular tissue, compared to patient matched proximal adjacent to carotid artery tissue, to investigate the phenotypic transitions of SMCs during the calcification process in humans. A total of 20 clusters were categorized from this analysis, and comparison between tissue types identified a greater proportion of T-cell and monocytes in the disease-associated tissue, whilst EC and fibroblast populations were higher in the control tissue. However, the largest population difference between control and diseased tissue was attributed to a macrophage-like SMC cluster, with enrichment analysis identifying gene expression related to inflammatory signaling, immune response, degranulation, and migration. Protein interaction analysis identified significant involvement of MMP9, CXCL8, SPP1, and LGALS3 among others, with upstream transcription factors such as NFkB1, RELA, SP1, JUN, and SPI all associated with downstream gene regulation.

Lin et al. (46) sought to identify SMC subpopulations which arise in response to intimal hyperplasia development through the development of a genetic mouse model of elastin insufficiency. Single-cell sequencing of cells isolated from the ascending aortas of these mice identified 14 distinct clusters, of which three were attributed to SMCs as defined by markers *Myh11, Acta2* and *Cnn1*, respectively. *Myh*11^+^ cells had the most significant expression of SMC marker genes, with *Acta*2^+^ cells exhibiting a more myofibroblast-like expression profile and *Cnn*1^+^ cells expressing genes related to cell proliferation. Subsequent pathway analysis indicated a closer transcriptional profile between the *Acta*2^+^ and *Cnn*1^+^ clusters, suggesting that *Myh*11^+^ SMCs are further differentiated. Reactome pathway analysis suggests a less contractile phenotype for the *Acta*2^+^ and *Cnn*1^+^ clusters, with the former exhibiting a fibroblast, ECM-synthesizing phenotype, and the latter a more proliferative phenotype. Validation experiments in immunostained tissue sections for cluster specific markers were able to localize these two subgroups specifically to the neointimal area.

Brandt et al. (50) undertook single-cell sequencing of CD45^−^ cells isolated from atherosclerotic aortas of *ApoE*^−/−^ mice on a normal and high cholesterol diet, with cells categorized based localization to atheroprone and atheroresistant regions (aortic arch and descending thoracic aorta, respectively). Differential regulation of genes was identified in the atheroprone cell cluster based on diet associated with apoptosis, inflammation, atherogenesis among others. In the atheroresistant cell groups, despite no detectable plaque formation, a high cholesterol diet still resulted in upregulation of genes related to an atherogenic stress response, including regulatory genes for SMC differentiation, apoptosis, and inflammation. Unsupervised clustering of a pooled cell population identified seven distinct CD45^_^ clusters—four of which were exclusive to the atheroprone region with gene expression indicating a foam cell-like, inflammatory, calcifying, phenotype transitional subgroups with loss of SMC contractile genes (*Myh11* and *Acta2*). Clusters specific to atheroresistant regions maintained and even increased contractile marker expression. In particular, the authors identified a significantly upregulated gene associated with the macrophagic/calcific phenotype—growth differentiation factor 10 (GDF10)—which was validated *in vitro* to modulate ossification, osteoblast differentiation and SMC phenotypic switching.

Quiles-Jimenez et al. (47) investigated the function of a DNA glycolase *Neil3*, associated with a role in atherogenesis, using a *ApoE*^−/−^*/Neil3*^−/−^ mouse model and NEIL3 knockout human aortic SMC cell culture. These mice had significantly increased atherosclerotic lesion areas, without changes in systemic lipid levels. Cell markers in the plaques remained similar to *ApoE*^−/−^ controls, but with greater SMC medial layer thickness and disorganization, and a clear phenotypic shift was identified in these cells with gene expression associated with increased proliferation, lipid-accumulation and de-differentiation. *In vitro* analysis revealed that NEIL3 deficiency is accompanied by a phenotypic shift to macrophage-like characteristics, with expression of markers CD68, TGFβ, and MMP2. Next-generation messenger RNA (mRNA)-sequencing identified gene enrichment for cell proliferation/apoptosis, differentiation, growth factor response, and others when compared to *ApoE*^−/−^ controls. This sequencing data, combined with proteomic analysis, revealed that Neil3-deficiency leading to SMC phenotype switching occurs *via* activation of the Akt signaling.

#### Macrophages

Six articles (22, 24, 29, 36, 49, 51) explored the contributions of macrophages to atherosclerosis and vascular inflammation. Four (22, 24, 36, 49) of these articles utilized single-cell sequencing exclusively, one (51) utilized next-generation microRNA sequencing, and the remaining paper (29) utilized a combination of single-cell and next-generation RNA sequencing methods.

Cochain et al. (22) utilized single-cell sequencing to explore the heterogeneity of aortic macrophages subsets within murine atherosclerosis. *CD45*^+^ cells were extracted from both non-diseased and atherosclerotic low-density lipoprotein receptor deficient (*Ldlr*^−/−^) male mice, and unsupervised clustering analysis revealed the presence of 13 unique cell clusters, five of which were exclusively expressed in atherosclerotic models. These included CD8^+^ T-cells, monocyte-derived dendritic cells (MoDC), monocytes and 2 distinct macrophage clusters. Macrophages comprised of three classes from this data: (1) traditionally activated (M1) macrophages expressing atherogenic markers such as chemokine receptor *CCL3* and *IL1B*; (2) M2 macrophages expressing anti-atherogenic markers including *F13A1* and *CCL24*; and (3) a novel, smaller subset with significant expression of *TREM2*. Gene ontology analysis of this cluster highlighted unique functions including lipid metabolism and cholesterol regulation, with expression resembling osteoclasts, indicating a potential role in plaque calcification.

Rahman et al. (24) using single cell sequencing methods tested the hypothesis that M2 macrophages in atherosclerotic plaques derive from newly recruited monocytes. This involved utilizing an aortic transplantation model to assess plaque regression in normolipidemic and *ApoE*^−/−^ mice against mice deficient in chemokine receptors *CCR2, CX3CR1* and *CCR5* involved in inflammatory (*Ly6C*^*hi*^) or migratory (*Ly6C*^*Io*^) monocyte recruitment. Their results provide strong evidence that plaque regression and inflammation resolution is dependent on the recruitment from the *Ly6C*^*hi*^ population, generally considered M1 macrophage precursors, and that *Ly6C*^*Io*^ macrophages are unable to fulfill this role. Single-cell sequencing and fate-mapping studies further indicated that polarization of these macrophages to an M2 phenotype is dependent on the action of *STAT6*.

Kim et al. (29) utilized CD45^+^ cells from murine atherosclerotic aortas of WT, *ApoE*^−/−^ and *Ldlr*^−/−^ mice, fed a four, eight or 12-week western diet, to investigate the transcriptomic profiles of foamy vs. non-foamy macrophages in the intima of diseased vessels. Unsupervised clustering analysis of single-cell sequencing data identified 11 distinct leukocyte subpopulations, which were subsequently categorized using a fluorescent lipid-labeling flow cytometry method capable of determining lipid-laden foam cells based on granularity. This approach suggested that most lipid-laden, foamy leukocytes originate from clusters with expression heavily indicative of macrophage origin. Next-generation RNA sequencing and gene set enrichment analysis of the foamy and non-foamy macrophage clusters revealed upregulation of markers in non-foamy macrophages corresponding to leukocyte recruitment and inflammation progression. In contrast, foamy macrophage expression markers were more closely correlated with lipid metabolism, lipid transport, and oxidative phosphorylation, suggesting an anti-inflammatory role.

Li et al. (51) identified a role for both matrix stiffness and ox-LDL in modifying the behavior of macrophages in healthy and diseased conditions. Matrix stiffness and ox-LDL were shown to increase adherence of macrophages, coupled with an increased inflammatory response (TNFα and IL1B). Macrophage mobility appeared to be increased in disease conditions, however, the presence of ox-LDL reduced this migratory capacity. Analysis of microRNA differential expression between healthy and disease states indicated upregulation of fatty acid synthesis, MAPK signaling, p53 signaling, and apoptosis.

Lin et al. (36) developed a genetic fate mapping approach specific to circulating CX3CR1^+^ macrophage precursors to assess their contributions in progressing and regressing atherosclerotic plaques. Single-cell sequencing identified 11 clusters of specifically myeloid lineage in a combined progression and regression dataset, which implied a wide range of macrophage activation states existed in these cells beyond the classical M1 and M2 definition, with activation states in greater number during atherosclerosis progression compared to regression. Three clusters were specific to the regression model, with one exhibiting a B-cell like phenotype (Ebf1^hi^Cd79a^hi^), and another with upregulation of heat shock proteins (HSP^hi^) suggestive of a protective role. Finally, one cluster had a distinctive transcriptomic profile enriched in cell cycle and cell proliferative genes, suggesting the existence of a self-renewing monocyte state existing within the inflamed tissue, rather than an immediate differentiation of these cells upon migration to the plaque.

Burger et al. (49) undertook single-cell sequencing of CD45^+^ cells isolated from descending thoracic aorta and aortic arch from *ApoE*^−/−^ mice on both a normal chow and high-cholesterol diet. Plaque formation predominated in the aortic arch tissue but was negligible in the descending thoracic aorta. Clustering was defined based on tissue type and diet, which revealed 12 distinct clusters—three clusters specific to the atheroprone aortic arch with expression of inflammatory monocyte/macrophage and resident macrophage related genes and five clusters specific to the atheroresistant descending thoracic aorta. Of note, one unique cluster of resident-like macrophages (*Lyve*1^+^ macrophages) which were shown to expand with atherosclerotic plaques progression, and that these cells exhibited a pro-osteogenic action, *via* their high expression of CCL24, which encouraged the phenotypic transition of vascular smooth muscle cells.

#### Stem/Progenitor Cells

Five articles (18, 23, 25, 30, 42) specifically address the role of stem/progenitor cells in atherosclerosis and vascular inflammation. Four (18, 23, 25, 42) of these articles utilize single-cell sequencing explicitly, whilst the remaining article (30) utilized next-generation RNA sequencing.

Tang et al. (18) used a combination of single-cell RNA sequencing, genetic lineage tracing mouse models, and cell fate mapping to identify a subpopulation of vascular stem cells—*sca1*^+^*PDGFRa*^+^—which generate *de novo* SMCs in the media of arteries following severe vascular injury. Interestingly, the data show that these cells only contribute to vessel repair in cases of severe injury, with cell fate mapping and wire-injury modeling indicating that these cells remaining complacent in homeostasis and following minor injury.

Kokkinopoulos et al. (23) explored the role of murine adventitial *sca1*^+^ cells in hyperlipidemia-induced atherosclerosis, by means of differential gene expression analysis of WT and *ApoE*^−/−^ mouse models. Sequencing and gene ontology enrichment depicted these cells to have expression characteristics representative of migratory and locomotive action, as well as cytoskeletal organization and endopeptidase activity, suggesting involvement in epithelial-to-mesenchymal (EMT) transition. Additionally, it seems that both LDL-bound and free cholesterol further enhance this migratory capacity as well as inhibiting their differentiation capacity to ECs and SMCs both *in vitro* and *in vivo*. This increased migratory capacity upon lipid loading appears to be the result of upregulation of microRNA (miRNA) *miR-29b*, which in turn induces *SIRT1* and *MMP9*. Combined, the authors suggest a direct link between adventitial progenitor cells function and blood cholesterol levels.

Gu et al. (25) was able to identify two distinct *sca1*^+^ clusters from single cell sequencing of perivascular adipocyte-derived stem cells (PV-ADSCs), the first of which demonstrated marker expression characteristic of endothelial cells (e.g., CD31 and Cadherin 5) with pathway association to VEGF receptor activity and PPAR signaling, indicative of angiogenic potential. Cluster two showed expression of markers associated with SMC differentiation, such as TGFβ signaling, PI3K-AKT signaling, PPAR binding and IGF binding. *Sca1*^+^ cells of the second phenotype were shown to significantly contribute to neointimal formation by differentiation toward a functional SMC phenotype in mouse vein graft models.

Steffen et al. (30) utilized next-generation RNA sequencing and PCR array analysis to scrutinize the identity of *sca1*^+^/*flk1*^+^ cells, which were hypothesized to exist as endothelial progenitor cells that are upregulated in response to endothelial injury. Mice were subjected to electrical injury to the left common carotid artery and sacrificed 5 days post-injury. Sequencing analysis of purified *sca1*^+^*/flk1*^+^ cells from these mice depicted expression levels highly comparable to regulatory B2-cells, including markers such as CD19, CD22, and CD79a/b. Additionally, surface markers such as CD1 and CD86 were shown to be highly expressed, as well as CD38 known to be specific to human regulatory B-cells, indicating that these cells exist as precursors to B2-like cells and not ECs.

Jiang et al. (42) utilized single-cell sequencing to investigate the exact identity and role of CD34^+^ cells using femoral artery tissue from WT C57BL/6J mice. This data was compared to lineage-traced Cd34-CreER^T2^; R26-tdTomato mice which underwent guide-wire injury, and the analysis revealed a heterogenous perivascular tissue population of CD34^+^ cells predominantly attributed to mesenchymal and EC origin. These cells were shown to contribute to endothelial regeneration and microvascular remodeling following injury. Bone-marrow transplantation experiments identified that the cells contributing to vascular repair are of non-bone marrow origin, with ablation of these cells aggravating adverse remodeling. Further single-cell sequencing of cells from the vascular injury model was undertaken, which revealed altered frequencies of cells at different repair timepoints in response to injury and marked changes in CD34^+^ subpopulations, such as SMCs and myofibroblasts of CD34^+^ origin. Pseudotime analysis of these cells, followed by cell differentiation experiments revealed a possible transition of adventitial CD34^+^ mesenchymal cells to ECs. Network analysis identified microRNA-21 as a negative modulator of the Smad7-pSmad2/3 pathway resulting in endothelial differentiation.

#### Endothelial Cells

Only two articles (32, 39) specifically explored the contributions of endothelial cells in atherosclerosis and vascular inflammation, both of which utilized next-generation RNA sequencing with one article (39) also opting to utilize the ChIP-Seq methodology.

Lai et al. (32) utilized a multi-time point approach to explore the effects of pulsatile shear (PS) and oscillatory shear (OS) stress on human primary ECs endothelial-to-mesenchymal (EndMT) genes expression. RNA sequencing of cells exposed to either PS or OS was undertaken at different time points over a 24-h period. Sequencing analysis of the PS-exposed cells showed an increasing expression of endothelial specific markers (e.g., CD31, vWF) over the 24-h period, whilst OS showed no significant change in EC marker expression. However, markers specific to mesenchymal cells were upregulated due to OS, including VCAM1 and SM22α, as well as inducing reactive oxygen species (ROS)-associated genes.

Zhou et al. (39) looked to explore how glucocorticoid receptors (GR) regulate ECs function using ChIP-Seq for primary mouse lung ECs under several conditions, including. control, GR siRNA-treated, glucocorticoid (dexamethasone) treated and a combination of these methods. These results showed significant binding close to the transcriptional start site, with 65 of the top 1,000 peaks showing both classic glucocorticoid responsive elements and *de novo* binding motif. RNA-Sequencing was undertaken which identified 231 glucocorticoid responsive genes and 203 genes differentially regulated by GRs. Comparison to GR ChIP-seq data in A549 cells revealed similar enrichment profiles for four main pathways: Wnt signaling, cytokine/chemokine signaling, angiogenesis, and cadherin signaling.

#### Lymphocytes

Three articles (19, 26, 38) investigated the contributions of lymphocytes in atherosclerosis and vascular inflammation, all of which utilized single-cell sequencing with one article (38) also opting to utilize next-generation RNA sequencing.

Sharma et al. (19) used several independent mouse models undergoing atherosclerotic plaque regression to illustrate an expansion in regulatory T-cell (Treg) populations compared to progressing and baseline plaques. Single-cell sequencing of CD45^+^ cells isolated from mice with progressive and regressive atherosclerosis depicted a greater proportion of thymus-derived Treg markers (e.g., NRP1) in progressing plaques, whilst regressing plaques expressed a higher proportion of markers representative of periphery induced T cells (e.g., Ly6A). Pathway analysis revealed that Treg cells in regressing plaques exhibited expression profiles associated with increased lymphocyte activation and increased metabolic activity, coupled with increases in genes such as TGFβ and IGF1, which were nullified by antibody mediated Treg depletion. Tregs were also shown to alter the macrophage landscape in regressing plaques, with increases in macrophage migration and death, coupled with a decrease in their proliferative capacity.

Winkels et al. (26) utilized single-cell sequencing to specifically elucidate the heterogeneous immune landscape of mouse atherosclerotic lesions, which revealed five distinct T-cell clusters, 2 B-cell clusters and a natural killer cell cluster. Interestingly, T-cells were revealed to accumulate predominantly in the lesion (alongside macrophages), whilst B-cells were localized to the surrounding vessel media and tissue. Comparison of chow and high fat diet mice revealed a transcriptional profile switch from a recruitment to pro-inflammatory phenotype, and mass cytometry analysis in response, particularly in the case of T helper cells (T_H_2) which expressed genes related to cytokine/chemokine expression and cell proliferation. Specific analysis of B-cell populations identified three distinct clusters, with the largest cluster (*CD43*^*high*^*B220*^*neg*^) associated with antigen presentation, cell adhesion and antibody generation. The *CD43*^*low*^*B220*^*high*^ cluster correlated with genes responsible for apoptosis and TNF-signaling, and the smallest cluster *CD43*^*neg*^*B220*^*high*^ related to cell division.

Wolf et al. (38) was able to identify the existence of a population of ApoB^+^ CD4^+^ T-cells in the lymph nodes of healthy C57BL/6 mice through a combination of *in silico* analysis, coupled with the development of a novel fluorochrome-coupled tetramer of recombinant MHC-II capable of binding and detecting CD4^+^ T-cells *via* flow cytometry. Interestingly, validation in both WT and *ApoE*^−/−^ mice revealed ApoB reactive memory T-cells are progressively activated in conditions of hyperlipidemia, and that their existence predates the onset of atherosclerotic disease. These cells appear to co-express markers associated with regulatory and effector T-cell phenotypes *via* flow cytometric analysis, and single-cell sequencing analysis identified a unique phenotype in ApoB^+^ cells which co-expressed regulatory and helper (T_H_1, T_H_17, and T_FH_) markers. Next-generation was subsequently undertaken in old and young *ApoE*^−/−^ mice, which identified a gradual phenotypic transition of ApoB-Reactive CD4^+^ regulatory T cells to a pro-inflammatory effector T-cell phenotype, which may be facilitated by the pro-inflammatory environment imposed by atherosclerosis. Further single-cell sequencing specific for aortic T-cells isolated from moderately (chow-fed) and severely (western diet) atherosclerotic *ApoE*^−/−^ mice. One predominant cluster (expressing markers of T_H_1 and T_H_17) appears to be selectively expanded with respect to disease severity.

### Tissue/Blood-Specific Sequencing

#### Atherosclerotic Plaque

Five articles (26, 34, 40, 43, 48) explored the total cellular and molecular composition of atherosclerotic plaques to identify heterogeneity within plaque areas and in comparison, to non-diseased controls. Three articles (26, 34, 43) utilized single-cell sequencing [one of which also utilized single-cell ATAC-Seq (34)], one article (40) utilized next-generation RNA sequencing and the last (48) used a combination of both methodologies. The work of both Winkels et al. (26) and Kan et al. (43) address atherosclerotic plaques but have been addressed in other more relevant sections of this review.

Depuydt et al. (34) undertook a combination of single-cell RNA and ATAC-Seq methods to explore the total cellular heterogeneity of atherosclerotic plaques in humans. Fourteen distinct populations were determined, including ECs and SMCs, and a host of immune cell subpopulations for T-cells, B-cells, and macrophages. ECs comprised four subclasses, three of which displayed expression markers indicative of activated endothelium with a role in inflammation progression and cell adhesion. The fourth subset displayed classical smooth muscle markers such as ACTA2 and MYH11, suggesting involvement in endothelial-to-mesenchymal transition (EndMT). Two subclasses of SMCs were also identified—the first with traditionally recognized SMC markers (e.g., ACTA2), whilst the other represented a synthetic class expressing COL1A1, MGP, and KLF4, which suggest a pseudo-macrophage phenotype. T-cell clusters were isolated to identify five CD4^+^ and three CD8^+^ clusters, distinguished by activation state. CD4^+^ T-cells ranged in gene expression, with two associated with cytotoxicity and pro-inflammatory pathways, two associated with a naïve phenotype, and the final with a classical regulatory role. Of the CD8^+^ clusters, one exhibits a terminal cytotoxic profile; one displays a quiescent phenotype and the last appears as an effector-memory subset. Macrophages comprised three subclasses, two of which had a M1 phenotype and were believed to be activation states of the same cell type based on how recently they were recruited. One class, expressing foam cell (e.g., MMP9) and pro-fibrotic markers (e.g., TREM2), also expressed ACTA2, suggesting a synthetic stage with gain of smooth muscle cell characteristics.

Bao et al. (40) looked to utilize next-generation RNA sequencing to define the transcriptomic profiles of stable and unstable atherosclerotic plaques in human carotid arteries. Analysis of the sequencing reads from these groups identified 202 mRNAs, 488 long non-coding RNAs and 91 circular RNAs differentially expressed between stable and unstable plaques. Gene ontology and pathway analysis identified these transcripts to have roles in cellular stress responses across both groups. With respect to the genes corresponding to plaque stability, these were identified as having functions related to the immune response, nervous system functions, hematological activity, and endocrine system synthesis and secretion. Comparative analysis of these transcripts to proteomic data generated this group highlighted five key correlated genes in unstable plaques—CD5L, S100A12, CKB, CEMIP, and SH3GLB1. CD5L encodes for CD5, primarily expressed by macrophages, and promotes M2 polarization. S100A12 is known to bind to RAGE and activate NF-kB and ROS pro-inflammatory signaling. CKB and CEMIP are targets of two long non-coding RNAs identified in this study, which play a role in energetic hemostasis and epithelial-mesenchymal transition, respectively. Finally, SH3GLB1 has been implicated in apoptotic and autophagy pathways, but no studies have directly assessed its function in atherosclerosis.

Newman et al. (48) investigated the cellular and molecular contributions responsible for the fibrotic cap formation. Using lineage-traced Pdgfrb^SMC−Δ/Δ^ and Pdgfrb^SMC−WT/WT^ mice, initial validation studies revealed a dependence of PDGFRB signaling for SMC to contribute to plaques composition, but that these plaques did not differ in lesion stability or area compared to their control in the absence of SMCs. Next-generation RNA sequencing of brachiocephalic artery lesions from these same mice revealed that absence of PDGFRB signaling results in pathway activation specific to substrate utilization and bioenergetics to maintain plaque stability. Single-cell sequencing of medial SMCs, medial and adventitial SMCs, and lesion cells identified seven SMC specific clusters, with general trends of traditional SMC marker loss (Myh11, Acta2, etc.) across several clusters but increased osteochondrogenic markers (Sox9, Trpv4, etc.), suggestive of a metabolic reprogramming of medial and lesion SMCs during plaque development. Follow-up studies identified a large proportion of *Acta*2^+^ cells in the fibrotic cap were derived from non-SMC origins, with markers related to EndMT and macrophage-to-myofibroblast transition (MMT).

#### Adventitial Tissue

Only two articles (21, 25) have explored the cellular and molecular contributions of adventitial and perivascular tissue in atherosclerosis, both of which utilize single-cell sequencing and arise from the same research group.

Gu et al. (21) utilized single-cell sequencing to elucidate the cellular composition of vascular adventitia, using both WT and *ApoE*^−/−^ mouse models exhibiting early-stage atherosclerosis. Unsupervised clustering analysis identified 15 distinct groupings, with most of these populations representing mesenchymal cells, macrophages, T-cells, B-cells, and innate lymphoid cells (ILCs). Six clusters were characterized as non-immune, with one cluster attributed to adventitial ECs and SMCs, respectively. The remaining four groups (I–IV) were classified as mesenchymal populations, including sca1^+^, CD34^+^, and Thy1^+^ marker-positive cells. Immune clusters comprised of two macrophage groups, identified as pro-inflammatory and resident. The resident cluster expressed the pro-atherogenic chemokine Pf4, suggesting a role in cell activation to hyperlipidemia, attracting immune cells. The follow-up study by (25) specifically investigated the sca1^+^ clusters identified as adipocyte-derived stem cells from their sequencing analysis, has been addressed earlier in this review.

#### Cardiac Tissue

Two articles (43, 44) specifically explore the cellular and molecular constituents of cardiac tissue using single-cell sequencing, with the first (43) investigating the heterogeneity of aortic tissue in healthy vs. disease states, and the second (44) addressing the impact of hemodynamic factors, specifically disturbed flow, on the composition of carotid arteries.

Kan et al. (43) undertook single-cell sequencing on ascending aorta samples from C57BL/6J mice fed a chow and high-fat diet to determine variations in cell composition and molecular heterogeneity between healthy and diseased aortas. Analysis of these results, once clustered, identified 10 major cell types—fibroblasts, SMCs, ECs, immune cells (B-cell, T-cell, macrophages, and dendritic cells), mesothelial cells, pericytes, and neural cells. In the high-fat diet groups, ECs clusters exhibiting markers characteristic of lipid-transport and inflammation, proliferation, and leukocyte-like properties across three clusters with all showing increased contractile gene expression. SMCs were clustered into five groups, with characteristics defined as synthetic (proliferative), contractile, fibroblast-like, and inflammatory in order of prevalence. Macrophages were clustered into four distinct groups, two of which were resident and had strong proinflammatory and proliferative properties; one blood-derived cluster with pro-inflammatory and ECM degradation characteristics; and one exhibiting a lymphocyte-differentiation and immune-effector profile.

Li et al. (44) addressed the effects of disturbed blood flow at the single-cell level using mice which underwent partial carotid artery ligation. Clustering analysis showed ten distinct cell clusters specific to disturbed flow compared to control: two EC clusters, one SMC, three macrophage, and four immune cell clusters. Of the two disturbed flow specific EC clusters, the first had enrichment of the gene Dkk2 (essential to angiogenesis) and was revealed to be a flow-sensitive cell state that may derive directly from physio-normal (laminar flow) EC phenotype. The other was enriched for CD36 and showed strong association with genes related to lipid metabolism and storage. The SMC cluster showed significant expression of Spp1, and osteoblastic marker, as well as further markers related to osteoblast differentiation, blood vessel remodeling, collagen biosynthesis, and arterial stiffness. Trajectory analysis implicated disturbed flow as directly influencing the prevalence of this phenotype in SMCs. Two macrophage clusters specific to disturbed flow had expression of markers Trem2 and Birc2, respectively. The first cluster expressed genes associated with chemotaxis and leukocyte migration, whilst the other had functions distinct to proliferation such as cell cycle DNA replication, chromatin segregation and RNA stabilization.

#### Blood

Two articles (33, 35) investigated blood constituents directly using microRNA sequencing, the first of which addressed the role of microRNAs in dyslipidemia and atherosclerosis risk, and the second identified the role of microRNAs in modulating macrophage heterogeneity.

Karere et al. (33) exposed two groups of baboons to a high cholesterol, high fat (HCHF) challenge diet—one group exhibiting low serum levels of LDL cholesterol and the other with high levels. Next-generation sequencing revealed 517 microRNAs from liver samples, 490 of which were identical to human microRNAs. Comparison of expression between baboon phenotypes indicated 20 novel baboon miRNAs in the low LDL cholesterol group and 29 in the high LDL group. Most of these microRNAs were not diet specific; however, there was increased quantitative expression of microRNAs in high LDL phenotype group in response to the HCHF challenge diet, suggesting diet plays some role in miRNA expression in response to dyslipidemia. Differential expression profiling revealed several unique microRNA expression profiles between the baboon phenotypes, including polycistronic regulation and polymorphism of microRNAs. Target prediction of the differentially expressed microRNAs identified 1,357 targets implicated in atherosclerosis and/or cardiovascular disease related genes, some of which exhibited contrasting or varying expression levels between the groups or in response to the HCHF challenge diet. These included LDLR and VLDLR for fatty acid metabolism, as well as ACVR1B—a receptor belonging to the TGFβ superfamily that is commonly implicated in atherosclerosis and inflammatory processes.

Li et al. (35) undertook microRNA sequencing of plasma exosomes isolated from human control subjects or subjects with significant (>50%) coronary artery stenosis. Three hundred and forty-two known microRNAs were identified, of which 14 (3 downregulated, 10 upregulated) were significantly and abundantly present in the disease samples vs. control. Determination of the targets of these differentially expressed microRNAs identified 38 differentially expressed mRNAs, and subsequent co-expression network analysis identified correlations between five monocyte/macrophage-related cell populations, six microRNAs and 10 mRNAs. RT-PCR validation of these markers showed downregulation of miR-4498 and upregulation of mRNAs CTSS, CCR2, and TREM2 in the disease group, and an inverse relationship was identified for miR-4498 vs. mRNA expression and stenosis severity. *In vitro* validation depicted macrophage uptake of circulating exosomes, and that uptake resulted in increases in the aforementioned mRNAs. Combined, this data suggests exosome-derived microRNAs exosomes may play a causal role in the polarization of macrophages to a chronic inflammatory phenotype.

### Risk of Bias and Quality Assessment

**Figure 3:**
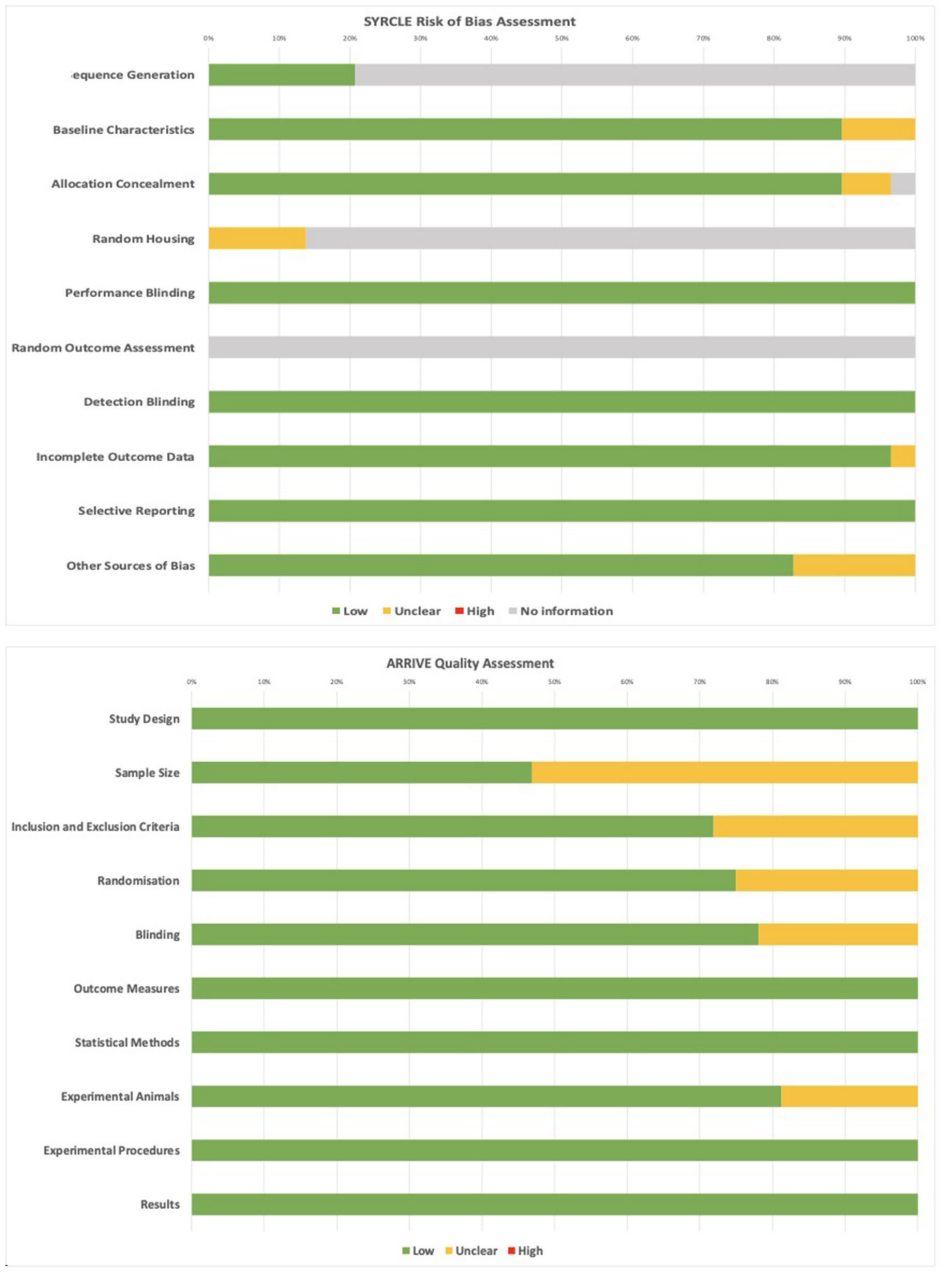
SYRCLE and ARRIVE assessments for quality and risk of bias of included studies.

Quality and risk of bias assessment was undertaken for all included articles using the ARRIVE checklist and SYRCLE risk of bias tools (Figures 3A,B). No studies were excluded from analysis based on these criteria. The most common limitations arising from the ARRIVE checklist was unclear information related to sample size calculation (18, 20, 22–24, 28, 30, 32, 33, 36, 38, 39, 43, 44, 46, 47, 49), blinding (18, 23–25, 31, 32, 41), randomization (20, 23, 24, 30, 31, 33, 34, 41), and specificity of inclusion/exclusion criteria (20, 23, 24, 28, 30, 31, 38, 40, 43). From the SYRCLE assessment, no information was provided regarding random outcome assessment in any of the included articles. Four articles provided unclear information about random housing, with the remaining articles providing no information (22, 26, 42, 43). Six articles had low risk of bias for sequence generation, with the remaining providing no information (19, 22, 26, 29, 42, 48). All other fields showed low or unclear bias for included studies, and factors such as blinding were all considered low due to the necessity for unblinded classification and analysis of sequencing data with respect to specific disease states, subjects, or cell types.

## Discussion

Atherosclerosis is well-established as one of the leading burdens to health globally. Despite many decades of research, there remains gaps in our understanding of the complex pathophysiology involved in the development and progression of this chronic inflammatory process. This can be attributed to the involvement of numerous cell types as well as a host of genetic, epigenetic, and environmental factors and the limitation of previously available methodologies for the study of the progression of the disease (1–3). Recent years have witnessed the emergence of new techniques that have contributed to our improved understanding of many complex diseases. In this study, we systematically reviewed studies that employed the techniques of single cell sequencing or/and next generation sequencing to develop better understanding of the processes involved in the development of vascular inflammation and atherosclerosis.

### Characteristics of Single-Cell and Next-Generation Sequencing Techniques

Single-cell and next-generation sequencing techniques offer significant yet differing means of further elucidating the onset and progression of atherosclerosis. Single-cell sequencing offers the capacity to examine the transcriptome profiles on a cell-specific level, allowing researchers to define cellular heterogeneity within their samples and determine properties inherent to that cell type (11). Such an approach facilitates the discovery of rare cell subpopulations and cell lineage analyses, as well as providing information on regulatory networks and differential gene expression within and between these cells. This technique involves the direct isolation of cells from a sample using microfluidics or flow activated cell sorting (FACS), a step that can present significant technical complexity in achieving the cell quality required for sequencing (52).

Next-generation sequencing, alternatively, is an overarching description for a series of advancements in genome sequencing technologies. In short, the advancements over traditional sequencing approaches include preparation of sequencing libraries without reliance on clonal amplification, and the spatially separated, amplified templates are sequenced in a massively parallel fashion such that thousands to millions of reactions can be undertaken simultaneously, offering comprehensive genome coverage in a faster and more sensitive manner (10, 53). Moreover, the sensitivity of this technology has been utilized to great effect, allowing researchers the opportunity to explore microRNAs, lncRNAs *via* rapid-amplification of cDNA ends (RACE)-sequencing, protein-DNA interactions using ChIP-Seq, and chromatin accessibility (ATAC-Seq) (54–57).

Whilst these technologies offer substantial improvements in both the accuracy and resolution of the transcriptomic data attainable, they are not without their drawbacks in their current state. Challenges facing the current utilization of single-cell sequencing methodologies have been comprehensively summarized by Lahnemann et al. (58), but in short, these challenges include: significant costs, complex computational analysis, lack of unified methodology from cell isolation to data interpretation, and high technical noise which favors highly expressed genes and masks weaker expression (59). Next-generation sequencing also presents some challenges, including a lack of sequencing localization, short read lengths (and the potential for read errors as a result) and a high dependency on reference datasets (60).

### Classification in Included Studies

Single cell sequencing found utility in 23 of the 34 included articles, despite greater complexity in operation and analysis, as well as the current technical and financial barrier to entry vs. more traditional sequencing approaches (58). Despite these challenges, its use in the articles reviewed has illustrated the flexibility and novelty of this technique, with research ranging from exploratory whole tissue and plaque composition analysis (26, 43), to investigation of unique cell phenotypes and their roles in disease (20, 22). In comparison, next-generation sequencing was utilized exclusively in a smaller subset of articles (*n* = 12), with this technique appearing to be specifically adopted for the validation and analysis of the role of pre-defined cell subsets or genetic targets (30, 31), as well validating the effects of biological and physical factors such as lipid levels and vascular hemodynamics and their contributions to disease progression (32, 33).

Of all cell types investigated in the included articles, SMCs featured predominantly (*n* = 12), with attention paid predominantly to their phenotypic switching capacity in disease states (20, 27, 28, 31, 37, 41, 45–47, 50). Macrophages were another prevalent focus of research (*n* = 7), with specific attention paid to their phenotypic heterogeneity in plaque progression and regression (22, 24, 29, 36, 49, 51). Stem/ progenitor cells were also commonly explored (*n* = 6), with attention paid to their presence and role in vascular adventitia, or more specifically as a source for vascular cell regeneration or repair (18, 23, 25, 30, 42). Comprehensive sequencing analysis of plaques was also frequently addressed (*n* = 5), with focus placed on the general composition and transcriptome of the plaque, as well as analysis of variations in expression between stable and unstable plaques (26, 34, 40, 43, 48). Little focus was placed on ECs and lymphocytes by comparison and may present an interesting area of note for future studies (32, 39).

### Advancements and Future Recommendations

In addition to single-cell and next-generation sequencing approaches, another unique approach has been described recently—spatial RNA sequencing (61). This technique offers researchers the ability to identify and quantify gene expression from tissue sections, localize that expression with near cell-specific accuracy and provide visual representation of the distribution of RNA transcripts within the section. This technology works by attaching tissue cryosections to spatial transcriptomic slides that have barcoded mRNA capturing reverse transcription primers on the surface. This binding facilitates the synthesis of complementary DNA that contains their unique barcode, allowing researchers to establish the specific tissue region that it originated (62). This technology, if implemented into current atherosclerosis research, could facilitate examination of diseased vessels under various physiological or pharmacological conditions, pinpointing transcript expression to specific lesion areas to determine their compositions and contributions to disease progression.

With increasing adoption of these techniques into research practice, considerations should be made on how best to utilize these methodologies. To date, there is no unified methodological approach best suited for single-cell sequencing, from cell isolation to data interpretation. On the information provided by the articles included in this review, there is significant heterogeneity in the methods of cell isolation and quality control, both of which are key steps in ensuring sequencing reads are representative of a significant, structurally, and transcriptionally intact cell population. Sequencing analysis is also heavily dependent on known cell biomarkers to classify clusters, and variations in pre-processing to account for these may result in significant clustering in some studies, and insignificant in others. To account for these variations, future studies should carefully consider the reproducibility of their workflow, and validation with other alterative sequencing methodologies could inform the reliability of their current approach.

The findings from this work and the implementation of these modern sequencing approaches into atherosclerosis research also provides a blueprint for translatability into other adjacent avenues of research, for instance into understanding the pathophysiology of vein graft disease. To date, no implementation of any of the aforementioned sequencing approaches have been utilized in elucidating the onset and progression of vein graft disease following coronary artery bypass surgery (CABG), despite its comparability to traditional atherosclerosis and its high prevalence and risk to a substantial patient population worldwide (63, 64). A comprehensive understanding of the pathophysiology of vein graft disease is lacking, particularly with respect to the impact on the phenotypes of vein graft cells and their interactions once implanted into a new hemodynamic environment. The use of single-cell, next-generation and spatial sequencing methodologies may be essential in improving our understanding of this disease and how preventative interventions can be developed for patients who have or will undergo CABG surgery in the future.

### Limitations

Whilst this is the first systematic review of the use of these sequencing methodologies in the study of atherosclerosis, it is not without its limitations. Given the heterogeneity of the research models, methods and outcomes of the included studies, a formal meta-analysis was not undertaken. Narrowing the scope of this review may have allowed for a comparative analysis to be undertaken, such as has been recently undertaken by Zernecke et al. (65) and Conklin et al. (66) in the comparison between sequencing datasets and between sequencing and *in vitro* models, however, these analysis approaches garner data which falls outside the scope of this review. Another limitation exists when comparing single-cell sequencing data between publications. Analysis of sequencing data involves complex bioinformatics analysis to denoise data, remove low quality reads and classify cell expression into groups based on known markers, and whilst packages such as 10 × Genomics CellRanger (67) exist to simplify this process, the specific criteria can vary between runs to optimize results. As such, novel cell clusters identified by one research group may be considered low quality reads by another and would thus be excluded.

### Summary

Single-cell and next-generation sequencing technologies can play a significant role in illuminating the functional role of different cells and subsets of cells in physiological and pathophysiological states. The continued implementation of these technologies, particularly as they become more readily available, may present significant opportunities to explain the exact pathophysiology of atherosclerosis to optimize prevention, management, and treatment. Concurrently, there exists a significant degree of translatability for these findings and sequencing approaches to other vascular complications such as vein graft disease, given the knowledge gap that currently exists in this area, prioritization of these techniques should be considered for any future research to advance the understanding of this disease.

## Data Availability Statement

The original contributions presented in the study are included in the article/Supplementary Material, further inquiries can be directed to the corresponding author/s.

## Author Contributions

LM, GM, and MZ: conceptualization and design. LM, SL, RA, GM, and MZ: analysis and interpretation. LM, SL, RA, and MZ: data collection. LM: writing the article and overall responsibility. LM, SL, RA, SG, M-SS, GA, GM, and MZ: critical revision and final approval. All authors contributed to the article and approved the submitted version.

## Funding

This work was supported by funding from the British Heart Foundation (CH/12/1/29419) to GM and SL, and the University of Leicester which provides funding matched to this BHF award to LM.

## Conflict of Interest

The authors declare that the research was conducted in the absence of any commercial or financial relationships that could be construed as a potential conflict of interest.

## Publisher's Note

All claims expressed in this article are solely those of the authors and do not necessarily represent those of their affiliated organizations, or those of the publisher, the editors and the reviewers. Any product that may be evaluated in this article, or claim that may be made by its manufacturer, is not guaranteed or endorsed by the publisher.
