# Supplementary material for: Next-Generation and Single-Cell Sequencing Approaches to Study Atherosclerosis and Vascular Inflammation Pathophysiology: A Systematic Review
Source: Front Cardiovasc Med. 2022 Mar 28;9:849675. doi: 10.3389/fcvm.2022.849675 (PMC8996078; doi:10.3389/fcvm.2022.849675)
Supplement: Supplementary file 1 [file Data_Sheet_1.docx]

**Systematic Review - Search Strategy**

Database originally searched on 13/10/20. Updated search undertaken on 15/02/22.

1. **Single-Cell + Next Generation Sequencing**

**Embase through OVID**

1. (scRNA-seq or (single-cell adj5 sequencing) or next-generation sequencing).ti,ab.

2. vascular inflammation.mp. or exp vasculitis/

3. atherosclerosis.mp. or exp Atherosclerosis/

4. 2 OR 3

5. 1 AND 4

**319 (original) 🡪 476 (updated)**

**Medline through OVID**

1. (scRNA-seq or (single-cell adj5 sequencing) or next-generation sequencing).ti,ab.

2. vascular inflammation.mp. or exp vasculitis/

3. atherosclerosis.mp. or exp Atherosclerosis/

4. 2 OR 3

5. 1 AND 4

**131 (original) 🡪 189 (updated)**

**Web Of Science through Clarivate**

1. AB = (scRNA-seq OR (single-cell NEAR/5 sequencing) OR (next-generation sequencing)) Indexes=SCI-EXPANDED, SSCI, A&HCI, CPCI-S, CPCI-SSH, ESCI Timespan=All year
2. ALL = (Atherosclerosis OR Arteriosclerosis) Indexes=SCI-EXPANDED, SSCI, A&HCI, CPCI-S, CPCI-SSH, ESCI Timespan=All years
3. ALL = (vascular inflammation OR vasculitis) Indexes=SCI-EXPANDED, SSCI, A&HCI, CPCI-S, CPCI-SSH, ESCI Timespan=All years
4. #2 OR #3
5. #1 AND #4

**225 (original) 🡪 362 (updated)**

**Original Results (13/10/2020) : 675 references -> 509 after duplicates removal**

**Updated Results (15/02/2022): 1027 references 🡪 791 after duplicates removal**
